# Mucinous adenocarcinoma of the appendix presenting with atypical symptomatology and presence of pseudomyxoma peritonei: a case report

**DOI:** 10.1186/1757-1626-2-9089

**Published:** 2009-11-25

**Authors:** Konstantinos Alexiou, Nikolaos Sikalias, Maria Demonakou, Sylvia-Christina Mylona, Vasileios Triantafyllis, Asimina Kalogirou, Georgios Antsaklis

**Affiliations:** 1 Department of Surgery, "Sismanoglio" General Hospital of Athens, (Sismanogliou 1), Marousi - Athens (15126), Greece; 2 Department of Anatomical Pathology, "Sismanoglio" General Hospital of Athens, (Sismanogliou 1), Marousi - Athens (15126), Greece

## Abstract

**Introduction:**

Primary tumors of the appendix are unusual and most of them are carcinoids. Their main presentation is that of an acute appendicitis or as a palpable mass, mainly in the right lower quadrant.

**Case presentation:**

A female patient with mucous adenocarcinoma of the appendix, which primarily presented as atypical abdominal pain. Diagnosis of the disease was made after appendicectomy and histopathological analysis of the specimen. The patient finally underwent a complementary right hemicolectomy.

**Conclusion:**

Mucin producing adenocarcinomas of the appendix are a category of rare cancers of the gastrointestinal tract. Although at present they are a well studied pathologic entity, the crucial issue of their preoperative diagnosis remains unsolved.

## Introduction

Primary tumors of the appendix are unusual and most of them (almost 85%) are carcinoids [1]. Adenocarcinomas of the appendix are a category of rare tumors of the gastrointestinal tract, with a frequency of 0,2% - 0,5% of all intestinal malignancies and 4% - 6% over neoplasmatic lesions of the appendix [2,3]. The first case of a primary adenocarcinoma of the appendix was reported by Berger on 1882[4]. Mucin-producing cystedenocarcinomas or mucous adenocarcinomas, and non-mucin producing or colonic type adenocarcinomas are included in this category.

The main presentation of these tumors is that of an acute appendicitis (30%-50%) or as a palpable mass mainly in the right lower quadrant [3]. Less frequently they may present in female patients as an ovarian tumor [4]. Nevertheless mucous adenocarcinomas are reported as having the greatest tendency among tumors to perforate, leading occasionally to the formation of pseudomyxoma peritonei (PMP)[3]. This specific image is met in 0,2% - 0,3% of all appedicectomies and comprises an uncommon and poorly studied situation with gelatinous ascitic collection and multiple mucous peritoneal implantations. In the case of benign cystadenoma, perforation causes the formation of localized collections, while in malignant adenocarcinomas mucous is spread and abundant [5]. Treatment of PMP comprises of surgical debulging of the peritoneal cavity, appendicectomy, omentectomy and bilateral oophorectomy in women. This treatment is proposed by different studies, showing that the primary location of the adenocarcinoma is almost exclusively the appendix and suggesting that ovarian carcinomas are in fact secondary metastases [1,3].

## Case presentation

**Figure 1:**
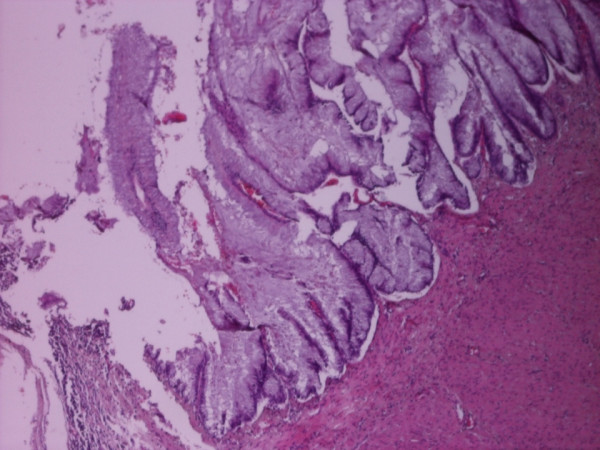
**(HEx10) A well differentiated mucous producing appendicial neoplasm without invasion of the stroma**.

**Figure 2:**
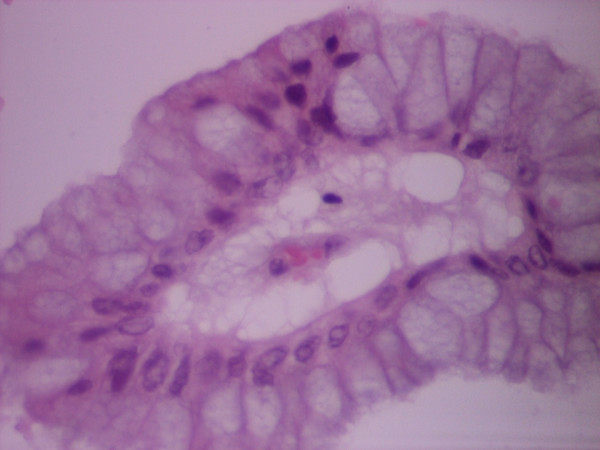
**(HEX40) Ville of the neoplasm covered by mucin producing cells with minimal atypia**.

A Caucasian 64-year old female Greek patient presented with hypogastric and right lower quadrant abdominal pain, which started 24 hours before, without other pathological findings in the clinical examination. Laboratory tests on admission showed: WBC: 7.200/mm^3 ^(with normal type), Hb:13,3 g/dl, Hct:40% and normal coagulation values. The patient was hospitalized for further investigation and underwent medical treatment for 6 days. Ultrasonography of the lesser pelvis showed a small quantity of free liquid located in the Douglas area and the right parametrium. Computed tomography of the pelvis reported normal internal organs (uterus, ovaries) and the patient was relegated for gynaecological evaluation after the initial clinical symptoms were minimized. One month later the patient presented again with the same abdominal pain. The diagnosis of acute appendicitis was set and the patient underwent an open laparotomy on a Mac Burney section. During the operation a dense and diffuse myxomatous collection was found around the appendix. Appendicectomy took place as well as thorough cleaning of the peritoneal cavity. Histological examination of the specimen revealed a mucous adenocarcinoma of the appendix [Fig 1, 2] which was classified as stage B (T_3_N_0_M_0_) on Duke's staging system (Astler Coller modification)[2]. The patient was then scheduled for a second operation and underwent a right hemicolectomy, which is proposed as the treatment of choice for this type of neoplasms. Since there was no evidence of synchronous presence of ovarian tumor, no additional oophorectomy was performed.

## Discussion

A retrograde study was performed concerning the appendicectomies that took place in General Hospital Sismanogleio over a ten year period between 1998 - 2008. A total of 2148 appendicectomies was performed (47,75% males, 52,25% females). Eleven (12) cases of primary appendiceal neoplasms were identified (frequency 0,37%), eight (8) of them being mucin-producing entities. Only one was positively identified as a mucous adenocarcinomas and right hemicolectomy with synchronous oophorectomy was performed as treatment of choice. Four (4) tumors of endocrine origin were recognized (frequency 0,19%) three (3) of them being benign carcinoids and one of uncertain classification. For this last case, right hemicolectomy was performed and diagnosis of the tumoral character was set after proper laboratory analysis. Carcinoids of the appendix are of low malignancy (> 90%, 5-year survival) compared to those developing in other organs and simple appendicectomy is sufficiently curative when their size is not over 1,5 cm. They rarely reach sizes over 2 cm and in this case they are considered to be malignant and right hemicolectomy is indicated [1]. Epithelial tumors of the appendix are not frequent and might be benign (adenomas) or malignant (adenocarcinomas), which further divide in mucous and non-mucous adenocarcinomas [1,6]. Concerning their etiology, mucous adenocarcinomas are generally accepted to arise in pre-existing cystadenomas[1,2]. In general, mucous adenocarcinomas have better prognosis than those of colonic type/non-mucous ones. Treatment of choise is considered to be right hemicolectomy.

Positive diagnosis for these tumors is generally set after their histologic identification; nevertheless criteria should be set for preoperative recognition, in order to perform the treatment of choice in the first place. Among imaging methods CT scan offers 95% sensitivity for carcinomas of the appendix. As reported in related studies of preoperative CT sections in patients diagnosed with appendiceal cancer, morphological changes and alterations in the dimensions of the appendix can guide the diagnosis. 100% of cases had evidence of altered morphology of the appendix and increased dimensions (max diameter 1,3 - 6,0 cm and max thickness of the appendiceal wall of 0,4 - 2.0 cm.)[7].

Moreover, the presence of a cystic mass may suggest the presence of cystadenoma or cystadenocarcinoma[8]. Nevertheless, since the usual presentation of the disease is that of acute appendicitis, the preoperative positive diagnosis still remains impossible[3].

## Conclusion

Mucin producing adenocarcinomas of the appendix are a category of rare cancers of the gastrointestinal tract. Although at present they are a well studied pathologic entity, the crucial issue of their preoperative diagnosis remains unsolved.

## Abbreviations

PMP: pseudomyxoma peritonei; WBC: white blood cells; Hb: Hemoglobin; Hct: hematocrit; CT: computed tomography; TNM: Classification of Malignant Tumours (Tumor size, lymph Nodes, and Metastasis).

## Consent

Written informed consent was obtained from the patient for publication of this case report and accompanying images. A copy of the written consent is available for review by the Editor-in-Chief of this journal.

## Competing interests

The authors declare that they have no competing interests.

## Authors' contributions

KA, NS, GA have had an equally substantial contribution to the clinical diagnosis, surgical management and post-op follow-up of the patient. MD and AK performed the histological examination KA, NS and MD performed the retrograde study, SCM and VT drafted the manuscript. NS and GA are guarantors of the paper. All authors read and approved the final manuscript.
